# Scalable Biofabrication: A Perspective on the Current State and Future Potentials of Process Automation in 3D-Bioprinting Applications

**DOI:** 10.3389/fbioe.2022.855042

**Published:** 2022-05-20

**Authors:** Nils Lindner, Andreas Blaeser

**Affiliations:** ^1^ BioMedical Printing Technology, Department of Mechanical Engineering, TU Darmstadt, Darmstadt, Germany; ^2^ Centre for Synthetic Biology, TU Darmstadt, Darmstadt, Germany

**Keywords:** automation, artificial intelligence, biofabrication, 3D-Bioprinting, manufacturing, Organs-on-Chips, process automation

## Abstract

Biofabrication, specifically 3D-Bioprinting, has the potential to disruptively impact a wide range of future technological developments to improve human well-being. Organs-on-Chips could enable animal-free and individualized drug development, printed organs may help to overcome non-treatable diseases as well as deficiencies in donor organs and cultured meat may solve a worldwide environmental threat in factory farming. A high degree of manual labor in the laboratory in combination with little trained personnel leads to high costs and is along with strict regulations currently often a hindrance to the commercialization of technologies that have already been well researched. This paper therefore illustrates current developments in process automation in 3D-Bioprinting and provides a perspective on how the use of proven and new automation solutions can help to overcome regulatory and technological hurdles to achieve an economically scalable production.

## 1 Introduction and Background

Tissue engineering has played an important role in the field of regenerative medicine and biomedical engineering for many years. Through the targeted interaction of cells, suitable carrier materials, and growth stimuli, it is possible to produce a variety of tissue substitutes (Nerem and Sambanis, 2007) as well as *in vitro* models for preclinical studies (Godbey and Atala, 2002; Hirt et al., 2014). Anthony Atala, for example, succeeded in producing a human bladder and implanting it in a patient as early as 1999 (Atala et al., 2006). However, traditional tissue engineering methods often depend on the use of molds and cores and are thus restricted in their geometric freedom. Moreover, these methods are labor intensive and difficult to reproduce, which is not practical in the context of an economically scalable production, neither it is regarding standardization in terms of governmental regulation guidelines.

A promising technology to enable reproducibility and scalability while maintaining high quality and standardization is 3D-Bioprinting. In conventional additive manufacturing processes, 3D-Printing is used for rapid prototyping on the one hand and to move from mass production to mass customization on the other (Bak, 2003; Berman, 2012). In contrast, the use of 3D-Bioprinting in combination with further automation steps is intended to enable scalable and standardized production of printed tissue or microfluidic models to replace labor intensive handcraft. It also provides the capability to use digital models that can be easily adapted to individual needs for functional human tissue substitutes or replicas such as liver, skin or bones. 3D-Bioprinting covers a wide range of different processes and technologies that differ fundamentally in terms of the method of transferring bioink from the cartridge to a substrate or a previously printed layer. Besides procedural differences 3D-Bioprinting methods offer some common significant advantages. They allow the use of sensor-based control and regulation of the printing process and thus enable online quality monitoring, which offers great added value in terms of achieving reproducible printing results with a high shape-fidelity, printing resolution and cell viability. Sensor integration and online *quality assessment* (QA) has the potential to pave the way for standardized manufacturing platforms. In conjunction with robotic process automation, this could enable the industrially scalable production of *clean meat* or *Organs-on-Chips* (OoCs) through 3D-Bioprinting. In addition, real-time recording of sensor data and corresponding online quality control can provide a foundation for the certification of *Advanced Therapy Medicinal Products* (ATMPs), which is critical for the approval of clinical applications.

Bioprinting technology can be looked at from two different perspectives, a rather technical one or an economical one. The former is subject of current research and technical advantages are widely observed. The economic consideration, however, is often disregarded. Therefore, online QA and further automation, suitability for scalable production of printed tissue and standardization are often ignored. This paper places current automation solutions in the context of scalable biofabrication, considers transferable processes from other industries, and provides a perspective on the fully automated use of 3D-Bioprinting processes to move from handcraft to standardized production.

## 2 Hardware, Sensors and Automation Potentials in 3D-Bioprinting

Increased automation of bioprinting processes, for example, enables online quality control and live adjustments, as well as scalability of the printing processes, leading to an improvement of the current state from both an economic and medical perspective. Thus, the following chapter presents an overview of the most common hardware components regarding already established bioprinting automation, future automation potentials and procedural interfaces for further automation as well as transferrable technologies from other industries.

## 2.1 Analysis of the Automation Potential of the Most Prominent Hardware Components

**FIGURE 1:**
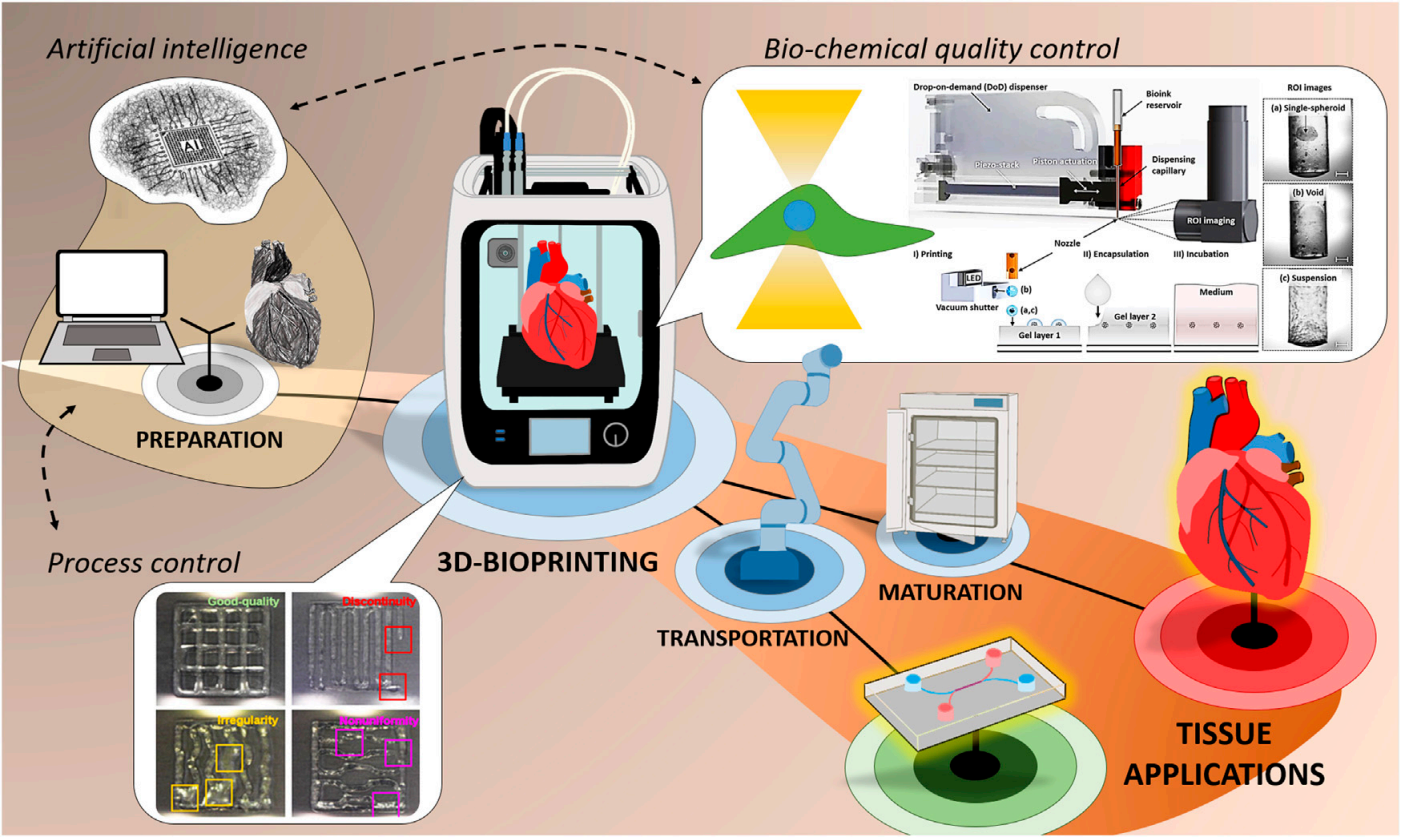
General 3D-Bioprinting process chain and illustration of hardware components with examples for the use of QA-Sensors towards live anomaly detection for process control (reprinted (adapted) with permission from (Jin et al., 2021). Copyright 2021 American Chemical Society) as well as the use of ROI imaging for bio-chemical quality control (Tröndle et al., 2019).

To identify automation potentials and find currently applied automation solutions, hardware components and process steps need to be observed and analyzed. Due to the wide range of different systems and printing methods those differ. Yet there are fundamental similarities that can be schematically illustrated cumulatively (Figure 1).

In general, the 3D-Bioprinting process chain can be distinguished in print preparation, the printing procedure, and post-printing tissue maturation (Figure 1). Print preparation covers the entire path from 3D design, to the generation of a data set for path calculation of the subsequent print path, to material synthesis and cell cultivation as well as the final bioink formulation. In the post-printing phase, the printed 3D structure is typically cultivated in an incubator, conditioned and is then available for further use, e.g., for OoCs, implantation or further examination of the tissue for research issues. Within the printing process which comprises fluid handling, robotics and control engineering to transfer bioink from the cartridge to a substrate or previously printed layer, a preparatory phase, the actual printing phase and the post-processing of the printed object can be subdivided in terms of automation. All these three phases offer automation potentials, and due to smooth transitions between the phases, not all components can necessarily be assigned to one phase. After hardware initialization and typically a system-depend cleaning protocol, the preparatory phase starts. There, the previously designed 3D data and the bioink are loaded into the printing system and the print cartridge, respectively. One or more bioinks are then deposited layer by layer according to the predefined print path. The way in which the material is applied and the shaping of the 3D structure are highly dependent on the specified bioprinting method and strategy (Duarte Campos and Blaeser, 2021). The most frequently used technologies and strategies are either dependent on traditional deposition techniques (extrusion- or inkjet-based) or optics-/light-based (stereolithography or laser-assisted) (Choudhury et al., 2018). Independent of the system, online process monitoring and control as well as QA-methods are taking on an increasingly important role during the printing process. The use of *Artificial Intelligence* (AI) for camera-based detection and evaluation of anomalies (Jin et al., 2021), bio-chemical quality control (Tröndle et al., 2019) and a monitoring system with direct print path feedback and correction (Armstrong et al., 2019) are just a few examples. AI could help to combine and accel those technologies even further. The post-processing phase includes all steps that take place after printing and before cultivation in the incubator, e.g. robotic transportation of the printed object and intermediate storage in a magazine for OoC-applications. Further examples of the current state and future potentials regarding the print-process automation are presented and discussed in the following chapters.

In addition to the general view and the former illustration (Figure 1), it is useful to get an overview of the different printing methods to connect them with hardware, sensors and control components. Table 1 shows in a structured way which process steps, components as well as sensors and actuators are applicable for which 3D-bioprinting method with regard to process automation.

**TABLE 1:** Assignment of process steps, involved printing elements and hardware components to their applicable printing methods.

Process Steps	Print Element	Components	Actuators	Sensors	Parameter	Applicable to	References
***(Un)loading and Transportation***	Substrate and/or printed structure	Gripper	Pneumatics	Pressure, Force	Weight, Surface texture	All	(Liu and Chiu, 2017; Wang and Hirai, 2018; Zhong et al., 2019)
			Adhesion	Force			
			Mechanics				
			Magnet				
		Robotics	Gantry robot	Position sensor/Potentiometer	Orientation/Position	All (depending on robotic concept)	(Li and Liu, 2019; Santoni et al., 2021; Tan et al., 2021)
				Accelerometer	Acceleration		
			Joint robot/Robotic-arm				
				Gyroscope/Rotation angle sensor	Orientation/Position		
	Bioink delivery	Microfluidic bioink supply	Valves (pneumatic, electromagnetic)	Flow, Pressure, Inductivity	Sensor-specific parameters, Viscosity, Cell viability	Inkjet, Microvalve-, Extrusion-based processes	(Chung et al., 2013; Ramiah et al., 2020)
		Spheroid-delivery	Pneumatic, mechanical	Optics, pressure	Spheroid size, pressure	Spheroid-based processes	(Moldovan et al., 2017; Ayan et al., 2020)
		Coating device	Squeegee	Optics	Cell Viability	Laser-based bioprinting	(Kačarević et al., 2018; Kingsley et al., 2019)
		Hose system	Cooling/Heating element	Temperature	Temperature	All	-
			-	Pressure	Pressure		
				Optics	Cell Viability		
		Pump/Compressor	Mechanical, Peristalic, pneumatic	Pressure, Temperature	Sensor-specific parameters	Inkjet, Microvalve-, Extrusion-based processes	Ozbolat and Hospodiuk, (2016)
***Printing***	Bioink dispenser	Reservoir	Mixer	-	-	Inkjet, Microvalve-, Extrusion-based processes	(Ng et al., 2017; Xu et al., 2019)
			Cooling/heating element	Temperature	Temperature		
			-	Optics (transmission, spectroscopy, microscopy)	Cell Viability		
				Pressure	Sensor-specific parameters		
				pH-sensor			
				CO_2_-sensor			
		Nozzle/printer head	Valves (pneumatic, electromagnetic)	Flow, Pressure, Inductivity	Sensor-specific parameters, Viscosity, Cell viability	Microvalve-based processes	Gu et al. (2018)
			Membranes	-	-	Inkjet	
			Cooling/heating element	Temperature	Temperature	All	
			Piezoelement	-	Voltage	DoD processes	
			Needle	-	-	Inkjet, Extrusion-based processes	
			Acoustics (no nozzle)	-	Voltage	Acoustic processes	
			Laser (no nozzle)	-	Voltage	Laser-based bioprinting	
		Light source/laser	Laser	-	Pixel size/resolution, Voltage	Stereo-lithography (SLA)	Kumar and Kim, (2020)
			Projector				
		QA-Sensors	-	Pressure	Sensor-specific parameters, Cell Viability, Droplet Size, Morphology, Shape fidelity, Number of Cells per unit	All	(Armstrong et al., 2021; Jin et al., 2021; Poologasundarampillai et al., 2021)
				Temperature			
				Volume			
				Optics			
	Build-up-3D-structure	Build plate/printing platform	Cooling/heating element	Temperature	Temperature	All	(Gómez-Blanco et al., 2021; Grigoryan et al., 2021)
			Level control	Level sensor, flowt, volume	Sensor-specific parameters	SLA	
				Optics	-		
			Incubator (nutrient supply)			All	
				CO_2_-sensor	CO_2_		
			Robotic axis	See above	See above	All	
***Cleaning***	-	Extraction system	Pneumatics	Pressure	Pressure	All	-
		Ultrasonic transducer	Ultrasonic transducer	-	Frequency		
		Water bath	Cooling/heating element	Temperature	Temperature		
		Scraper	-	-	-		
***Storage***	Substrate and/or printed structure	Incubator	Cooling/heating element	Temperature	Temperature	All	-
			Ventilation	CO_2_-Sensor	CO_2_		
			-	Optics, pressure, flow	Sensor-specific parameters		

The assignment shown here is not complete due to clarity reasons, but reflects the most important elements and a large part of the sensors and actuators for 3D-Bioprinting. Components that appear repeatedly in different print elements are listed only once, including their associated sensors and actuators. This provides a reasonable reference in a clear framework to the topic of automation and automation potentials. In addition to the above, from a technological cell biological point of view, further consideration of biosensors would be interesting, for example, in order to be able to draw conclusions about cell expression and contraction as well as proliferation and metabolic activities. Equally, from a manufacturing perspective, control and regulation, further sensors, e.g. for recording micro-vibrations, or safety-relevant elements such as end stops can be considered.

### 2.2 Currently Applied Sensor and Automation Concepts in 3D-Bioprinting

The term 3D-Bioprinting first emerged in scientific articles in the early 2000s, while the first bioprinter according to today’s understanding, a modified standard inkjet printer, originates from 2003 (Wilson and Boland, 2003). The first patents on bioprinting appeared in a similar time frame between 2001 and 2003 (Bicudo et al., 2021). Since its beginnings, the technology has already evolved greatly, advancing from a niche technology further and further into a wide variety of sectors to fulfill prerequisites for commercial applications (Combellack et al., 2018). During this development, automation is also becoming increasingly important in the context of process-driven research of 3D-Bioprinting in order to meet standardized, scalable and economic constraints (Santoni et al., 2021). Modern 3D-Bioprinting itself requires a certain level of automation to be functional, which leads to the current state of the art. The use of basic sensors, actuators and robotics as well as simple process monitoring measures is evident from the description of the different processes (Vanaei et al., 2021). Recent publications even already show single advanced technologies relying on QA methods for process prediction and adaptation (Rimann et al., 2016; Narayanan et al., 2018; Riba et al., 2020; Armstrong et al., 2021; Elbadawi et al., 2021). The goal of the latest research and development by universities, bioprinting companies, and companies in the life science and food industries are all-in-one platforms. These are designed to enable the use of multiple materials, tools for further processing and process monitoring, and the use of AI (Santoni et al., 2021). The described advances are mostly in the early stages and not ready yet for commercialization on a large and economic scale.

### 2.3 Most Promising Sensor and Automation Solutions from other Industries to Optimize 3D-Bioprinting

In other industries process automation is well known and broadly applied. It is also a well-researched subject area itself that is still constantly evolving. The following subsections present learncases from other industries that offer great potential to be applied to 3D-Bioprinting automation in the future. Of course, the elements and technologies use to overlap in the individual sectors, which is why hereafter the most advanced and most frequently used are mentioned in each case.

### 2.3.1 Food Industry

In the food industry, process automation solutions have been used in a variety of ways for many years. Gripping and transport systems as well as monitoring and ensuring a sterile working environment have a major role in the standardized and automated implementation of these measures (Ilyukhin et al., 2001; Holmes et al., 2013). For example, in large-scale processing of fruits and vegetables on plantations, non-invasive vacuum gripping systems are often used for fast and reliable pick and place applications to manipulate products and enable an end-to-end process chain (Blanes et al., 2011; Morales et al., 2014). To ensure high product quality and undamaged products, camera-based systems are used in combination with actuators that, for example, perform an automated sorting process (Bee and Honeywood, 2003; Bader and Rahimifard, 2020). In fully automated operations this is followed by direct packaging of the products according to specified standards. The processing of many foods requires a clean and uncontaminated atmosphere, for example to protect against the presence of salmonella. To meet the specified regulations, various methods are used here depending on the application (de Alwis and Fryer, 1990; Guzel-Seydim et al., 2004; Oscar, 2005; Huang et al., 2008; Velugoti et al., 2011).

### 2.3.2 Conventional Printing and 3D-Printing

Conventional printing processes are multiparametric and highly dynamic processes. For this reason, the use of sensors, actuators, mechanics and a control unit, which represents the structure of a mechatronic system (Tehrani et al., 2016), is highly advanced here in order to be able to achieve high and consistent print quality. Technologies for measuring and adjusting pressure, position, temperature and air bubbles play a crucial role in industrial printing applications and thus are broadly applied. To achieve better results, increase productivity and reduce downtimes, artificial intelligence methods are emerging more and more in modern industrial printing machines (Neeb et al., 2019).

In terms of its characteristics and general structure, conventional 3D-Printing is very similar to 3D-Bioprinting. This allows a good transfer of knowledge between the two technologies, so that 3D-Bioprinting can benefit from the more advanced knowledge of 3D-Printing. The focus here is specifically on material deposition (MacDonald and Wicker, 2016; Lee et al., 2017), robot kinematics (Qian et al., 2018; Jinghua et al., 2020), printing space and platform (Sitthi-Amorn et al., 2015), optical QA-methods and a fully digitized process chain or 3D-Printing factory (Rengier et al., 2010).

### 2.3.3 Artificial Intelligence

Artificial intelligence is entering everyday life more and more and offers a variety of innovative and useful methods to solve problems in an automated and intelligent way. Using machine learning, fast and precise predictions can be made about process parameters and results, and multi-dimensional sensor signals can be merged and interpreted. In modern industrial production facilities, sensor data fusion and predictive maintenance are already being used in the field to compare target and actual data and have proven to be accelerators here (Aliustaoglu et al., 2009; Hashemian, 2011; Indri et al., 2019; Nazir and Shao, 2021; Pech et al., 2021). Nowadays, medicine and medical technology use image processing methods to segment and classify structures in order to draw conclusions about diseases and the resulting therapies (Liu et al., 2019; Shi et al., 2021).

## 3 Discussion

### 3.1 Potential Impact on 3D-Bioprinting

Between 2016 and 2021 the number of publications on the topic automation in bioprinting and tissue engineering indicated a strong increase in interest^1^. Individual publications show groundbreaking results on how the use of automation solutions can help to monitor and adjust crucial process parameters and to draw conclusions about cell biological characteristics or allow the printing of complex 3D structures with hydrogels (Hinton et al., 2015; Shi et al., 2018; Armstrong et al., 2019; Jin et al., 2021; Poologasundarampillai et al., 2021; Yang et al., 2021). At the same time, the number of patents and companies embracing 3D-Bioprinting continue to grow (Santoni et al., 2021). However, no technology has yet achieved the major breakthrough to commercial marketing and industrial production for a broad mass (Ng et al., 2019). One reason for this is the lack of interfaces between different process steps, respectively the frequent interruption of the process chain by human intervention for transport or inspection tasks. Application-specific placement and transport systems, as often used in the food industry (chapter 2.3.1), can act as an interface and close the chain in a relatively simple way, thus enabling, for example, large-scale production of OoCs. Even in 2011 a publication showed the deficiencies of the simple use of 3D-Bioprinters and argued with the requirement of an additional sophisticated production line to enable commercial biofabrication (Mironov et al., 2011). The smart use and aggregation of sensor data is another way to facilitate the commercial market entry of 3D-Bioprinting. Combined with AI applications, this allows real-time data to be analyzed, predictions to be made, and even parameter adjustments to be made automatically. This not only leads to higher process accuracy and productivity, but in the future can become the foundation for standardization and therefore also the driving force on the path to certification to meet regulatory guidelines for medical therapies or food products (Li and Faulkner, 2017; Haeusner et al., 2021; Schmidt et al., 2021). Current studies predict, for instance, that clean meat could become part of the everyday diet in a few years (Chriki and Hocquette, 2020; Lee et al., 2020). Automation technology will play a major role in making the transfer from laboratory applications to scalable production and economically viable commercialization, thus exploiting the technological potential of 3D-Bioprinting as far as possible.

### 3.2 Challenges

Despite recent developments and an increased interest to use the power and knowledge of process automation to advance 3D-Bioprinting, the field has still a lot of room for further improvement. Within the nature of a multiparametric process with high standards towards every parameter and their combination, 3D-Bioprinting is a rather complex application. In addition to the high technological demands on the printing system and the materials for implementing the actual procedure, this places equally high standards on process automation and technological development for process optimization. This results in strict regulatory requirements for the approval of products, high research expenditure and high costs. To overcome these challenges multidisciplinary research should be conducted by experts in cell biology, pharmacy and medicine, but equally by engineers with knowledge in additive manufacturing, material science, artificial intelligence and mechatronics.

## 3.3 Conclusion and Outlook

3D-Bioprinting, whether in the field of new regenerative medical products as biomimetic and bionic cell-loaded implants, the development of clean meat or for drug discovery via the use of OoCs, has emerged as a novel innovative technology with great potential for the future. This is demonstrated both by the recent strong increase of interest from scientific institutions via rising publication numbers as well as exponential increasing patent registrations and industrial interest. Further research in the field of 3D-Bioprinting process automation, especially in the area of live quality monitoring, is essential to overcome technological and regulatory challenges. Progress from this and further developments in AI will be supported in the future by the smart combination and use of already known sensor and control technologies that can be burrowed from other sectors (e.g. food industry, conventional printing and additive manufacturing industry) to fully exploit the great potential of 3D-Bioprinting. Thus, the production of new regenerative medicinal products, clean meat and OoCs for animal-free drug development can be further advanced to make commercial biofabrication more realistic from an economic and scalability perspective.

## Data Availability

The original contributions presented in the study are included in the article/Supplementary Material, further inquiries can be directed to the corresponding authors.

## Footnotes

1 According to PubMed, publications on the terms “bioprinting automation” and “tissue engineering automation” have increased from 20 in 2016 to 47 in 2021 per year, accessed January 2022.
